# Annals of General Psychiatry

**DOI:** 10.1186/1744-859X-4-3

**Published:** 2005-02-01

**Authors:** Konstantinos Fountoulakis

**Affiliations:** 1 Aristotle University of Thessaloniki, Greece; 2 Deputy Editor, Annals of General Psychiatry

## Abstract

Our regular readers will notice that the title of our journal has changed from Annals of General Hospital Psychiatry (AGHP) to Annals of General Psychiatry (AGP) since January 1^st^, 2005. This was judged as necessary, in order to be able to serve better the aims of the journal. Our initial thoughts were that including the term 'General Hospital' in the journal's title would help us to launch a journal dedicated to the idea of Psychiatry as a medical specialty. But they were not justified; so, now the Annals of General Psychiatry (AGP) is born! It is still an Open Access, peer-reviewed, online journal covering the wider field of Psychiatry, Neurosciences and Psychological Medicine, and aims at publishing articles on all aspects of psychiatry. Primary research articles are the journal's priority, and both basic and clinical neuroscience contributions are encouraged. The AGP strongly supports and follows the principles of evidence-based medicine. AGP's articles are archived in PubMed Central, the US National Library of Medicine's full-text repository of life science literature, and also in repositories at the University of Potsdam in Germany, at INIST in France and in e-Depot, the National Library of the Netherlands' digital archive of all electronic publications. We hope that the change in the journal's name will cure the confusion caused by its previous title and help to achieve the journal's aims and scope, that is to help the world-wide promotion of research and publishing in the mental health area.

## 

Since January 1^st^, 2005 the title of our journal has changed from Annals of General Hospital Psychiatry (AGHP) to Annals of General Psychiatry (AGP).

The AGHP was launched in September 2002 as an Open Access, peer-reviewed, online journal covering the wider field of Psychiatry, Neurosciences and Psychological Medicine. It was aimed at publishing articles on all aspects of psychiatry.

However, it seems that the title caused a certain degree of confusion. As a reflection of this confusion, we received a lot of questions from colleagues asking whether their paper would fit the journal or not. These colleagues had authored general psychiatry papers, definitely inside the scopes of the journal, but their impression was that the journal, because of its title, had to do only with 'psychological medicine', 'psychosomatic medicine', 'consultation-liaison psychiatry' and similar areas, and not with general psychiatry as part of the medical model and tradition.

Repeated efforts from our side to change this impression were largely unsuccessful. So, changing the title seemed to be the only solution left in order to cure this misunderstanding.

So, now the Annals of General Psychiatry (AGP) is born! The web site has changed to . Our editorial board has been enlarged to include eminent figures from the field of Psychiatry. It is still an Open Access, peer-reviewed, online journal covering the wider field of Psychiatry, Neurosciences and Psychological Medicine, and aims at publishing articles on all aspects of psychiatry. Primary research articles are the journal's priority, and both basic and clinical neuroscience contributions are encouraged. The AGP strongly supports and follows the principles of evidence-based medicine.

The AGP is not a new journal. Instead, it is the continuation of the AGHP. It emphasizes a biopsychosocial approach to illness and health, and where possible a wider dialogue will be opened on specific articles thereby placing the research into a wider framework. Online publication with Open Access, are powerful tools to achieve these goals.

AGP continues to be published by BioMed Central, an independent publisher committed to ensuring peer-reviewed biomedical research is Open Access. That means it is freely and universally accessible online, it is archived in at least one internationally recognized free access repository, and its authors retain copyright, allowing anyone to reproduce or disseminate articles, according to the BioMed Central copyright and license agreement. AGP takes this a step further by ensuring that all published articles are Open Access.

AGP's articles are archived in PubMed Central, the US National Library of Medicine's full-text repository of life science literature, and also in repositories at the University of Potsdam in Germany, at INIST in France and in e-Depot, the National Library of the Netherlands' digital archive of all electronic publications.

BioMed Central is working closely with the Institute for Scientific Information to ensure that citation analysis of articles published in AGP will be soon available. Also, by being part of BioMed Central, AGP is a member of the Committee on Publication Ethics (COPE).

Recently both the BioMed Central and the AGP joined the World Health Organization Department of Mental Health and Substance Abuse initiative and signed the January 2004 joint Statement which was published in the AGHP [1]. This gives the AGP a new additional role, that is to contribute to the efforts of the WHO to galvanize mental health research and publishing in middle and low-income countries.

We hope that the change in the journal's name will cure the confusion caused by its previous title and help to achieve the journal's aims and scope, that is to help the world-wide promotion of research and publishing in the mental health area.

## Competing interests

Dr KN Fountoulakis has received support for research and educational reasons as well as honoraria and financial assistance with registration, accommodation, and/or travel expenses for conferences and meetings from Eli Lilly, Janssen-Cilag, AstraZeneca, Pfizer, Sanofi-Synthelabo, Novartis, Wyeth, Organon and Bristol-Myers Squibb.
